# Role of the gp85/Trans-Sialidases in *Trypanosoma cruzi* Tissue Tropism: Preferential Binding of a Conserved Peptide Motif to the Vasculature *In Vivo*

**DOI:** 10.1371/journal.pntd.0000864

**Published:** 2010-11-02

**Authors:** Renata R. Tonelli, Ricardo J. Giordano, Elena Magda Barbu, Ana Claudia Torrecilhas, Gerson S. Kobayashi, Robert R. Langley, Wadih Arap, Renata Pasqualini, Walter Colli, Maria Júlia M. Alves

**Affiliations:** 1 Departamento de Microbiologia, Imunologia e Parasitologia, Universidade Federal de São Paulo, São Paulo, Brazil; 2 Departamento de Bioquímica, Instituto de Química, Universidade de São Paulo, São Paulo, Brazil; 3 David H. Koch Center, The University of Texas MD Anderson Cancer Center, Houston, Texas, United States of America; 4 Department of Cancer Biology, The University of Texas MD Anderson Cancer Center, Houston, Texas, United States of America; New York University School of Medicine, United States of America

## Abstract

**Background:**

Transmitted by blood-sucking insects, the unicellular parasite *Trypanosoma cruzi* is the causative agent of Chagas' disease, a malady manifested in a variety of symptoms from heart disease to digestive and urinary tract dysfunctions. The reasons for such organ preference have been a matter of great interest in the field, particularly because the parasite can invade nearly every cell line and it can be found in most tissues following an infection. Among the molecular factors that contribute to virulence is a large multigene family of proteins known as gp85/trans-sialidase, which participates in cell attachment and invasion. But whether these proteins also contribute to tissue homing had not yet been investigated. Here, a combination of endothelial cell immortalization and phage display techniques has been used to investigate the role of gp85/trans-sialidase in binding to the vasculature.

**Methods:**

Bacteriophage expressing an important peptide motif (denominated FLY) common to all gp85/trans-sialidase proteins was used as a surrogate to investigate the interaction of this motif with the endothelium compartment. For that purpose phage particles were incubated with endothelial cells obtained from different organs or injected into mice intravenously and the number of phage particles bound to cells or tissues was determined. Binding of phages to intermediate filament proteins has also been studied.

**Findings and Conclusions:**

Our data indicate that FLY interacts with the endothelium in an organ-dependent manner with significantly higher avidity for the heart vasculature. Phage display results also show that FLY interaction with intermediate filament proteins is not limited to cytokeratin 18 (CK18), which may explain the wide variety of cells infected by the parasite. This is the first time that members of the intermediate filaments in general, constituted by a large group of ubiquitously expressed proteins, have been implicated in *T. cruzi* cell invasion and tissue homing.

## Author Summary

Chagas' disease, caused by the protozoon *Trypanosoma cruzi,* is an ailment affecting approximately 12–14 million people in Iberoamerica and is becoming increasingly important in North America and Europe as a result of migratory currents. The parasite invades mainly cells of the heart or the walls of the digestive tract. The patients with symptoms develop heart disease or gastrointestinal motor disorders. We and others have implicated the *T. cruzi* gp85/trans-sialidase surface protein family in the attachment of the parasite to the host cells. These proteins share a peptide motif called FLY. The involvement of FLY in parasite interaction with endothelial cells from different organs has been studied using bacteriophages expressing the FLY peptide as surrogates. We found that phages expressing FLY bind to endothelial cells in an organ dependent manner, particularly in the heart. Also, this peptide binds strongly to intermediate cell filaments, like cytokeratins and vimentin. These results indicate that FLY might be an important contributor to tissue tropism. It also supports the notion that the vasculature and the endothelial cells are important players in Chagas' disease. These data may have important implications in the pathology of Chagas' disease and novel therapeutic approaches for patients afflicted with this disease.

## Introduction

*Trypanosoma cruzi*, the causative agent of Chagas' disease, is an obligatory intracellular parasite that uses mammalian host cells to replicate and escape from the immune system to continue its life cycle [1]. The infective trypomastigote form is transmitted to humans (and other vertebrate hosts) by blood-sucking insects or blood transfusion, and recently recognized, by ingestion of infected triatomine contaminated food. Following infection, patients enter an acute phase of the disease in which parasites are often found in circulation, resulting in the infection of many tissues and organs. However, in a few weeks parasites vanish from the blood and patients enter the indeterminate or chronic stages of the disease. The reasons why some enter an indeterminate stage and never develop any symptoms while others evolve to the chronic manifestations of Chagas' disease is unknown. About two thirds of patients with symptoms will develop heart disease and the remaining ones gastrointestinal motor disorders, mainly a result of enteric nervous system injury caused by the parasite [2], [3], [4]. Bladder and other urinary tract disorders have also been described but much less frequently [2], [5]. The exact mechanism for such tissue and organ preference is still a matter of debate and research. Therefore, unveiling the mechanism of parasite cell attachment and tissue tropism is an important milestone to comprehend how the disease develops and progress.

Toward this end, our group and others have identified and studied a supergene family encoding the cell membrane glycoproteins known collectively as gp85/trans-sialidase, which involvement in trypomastigote cell entering has been well demonstrated [1], [6]. The gp85/trans-sialidases are not expressed by the noninfective epimastigote form of *T.cruzi*. There are approximately 700 gp85/trans-sialidase genes and equal amounts of pseudogenes, in the parasite genome of *T. cruzi*, Cl Brener strain [7] and the proteins encoded by this supergene family are believed to participle in parasite host cell adhesion and invasion by interacting with multiple ligands, such as laminin [8], fibronectin [9], collagen [10], [11], cytokeratin (CK) [12] and probably, other cell surface and extracellular proteins [1]. Members of the gp85/trans-sialidase family share a signature conserved sequence (VTVxNVxLYNR) upstream from the carboxyl terminus [13], [14]. It has been shown that a peptide encoding this conserved sequence (FLY, for short) promotes cell adhesion by binding to the intermediate filament protein cytokeratin-18 (CK18) [12]. FLY-promoted CK18 dephosphorylation and ERK1/2 signaling cascade activation, significantly increasing parasite entry into mammalian cells [15].

In the present study, it is hypothesized that the FLY peptide cell adhesion properties could also have an important role in parasite interaction with the vasculature *in vivo,* involving this peptide in parasite tissue tropism. Inhibition of gp85/trans-sialidases with monoclonal antibodies or anti-sense oligonucleotides block cell invasion by *T. cruzi,* suggesting that these proteins contribute to tissue infection [16], [17]. To investigate whether FLY participates in endothelial cell interaction and tissue homing, a combination of endothelial cell immortalization and phage display methodologies was employed. Tissue-specific microvascular endothelial cell lines from the *H-2K(b)-tsA58* mouse (termed ImmortoMouse) were used; this cell culture system was initially established to examine factors regulating angiogenesis and tumor cell arrest in different organ systems [18] and it has been successfully utilized to screen and to identify tissue homing peptides [19]. These endothelial cells carry a temperature sensitive SV40 large T antigen under the major histocompatibility complex *H-2K^b^* promoter and are conditionally immortal when cultured under permissive temperatures [20], [21]. Of note, when cultured *in vitro* these cells retain at least some of the molecular expression profile displayed *in vivo* [19], [21]. Combined with phage display [22], which is a powerful tool to interrogate the vascular expression profile and more importantly, tissue accessibility [23], [24] these methodologies constitute an experimental framework for discovery and validation of vascular-directed receptor-pair ligands [25]. Using that strategy, it is shown here that the FLY domain has a preference for the vasculature of certain organs, results which might have important implications in Chagas' disease pathology and progression.

## Materials and Methods

### Reagents, recombinant proteins and synthetic peptides

Anti-fd bacteriophage antibody (Sigma Aldrich), recombinant cytokeratin 18, cytokeratin 8, cytokeratin 20 and vimentin were commercially obtained (Cell Sciences). Peptides were obtained by the solid-phase peptide synthesis strategy as previously described [12] using the Fmoc-procedure in an automated bench-top simultaneous multiple solid-phase peptide synthesizer (PSSM 8 system from Shimadzu, Tokyo, Japan).

### Cell culture

Organ derived endothelial cells were cultured and propagated in DMEM medium (Invitrogen) supplemented with 10% fetal bovine serum at 33°C in 5% CO_2_ as described [21]. For the cell binding assays, the endothelial cells were first returned to the quiescent state by culturing at 37°C for 48 h.

### Phage mutagenesis

Phage displaying the FLY (VTVTNVFLYNRPLN) or FAY (VTVTNVFAYNRPLN) peptide sequences were cloned in the fUSE5 vector as previously described [22], [26]. Briefly, 500 ng from the synthetic oligonucleotides (FLY: 5′-CAC TCG GCC GAC GGG GCT AGC GTG ACC GTG ACC AAC GTG TTT CTG TAT AAC CGC CCG CTG AAC GGG GCC GCT GGG GCC GAA-3′; FAY: 5′-CAC TCG GCC GAC GGG GCT AGC GTG ACC GTG ACC AAC GTG TTT GCG TAT AAC CGC CCG CTG AAC GGG GCC GCT GGG GCC GAA-3′) were converted to dsDNA by PCR with the primer set 5′-TTC GGC CCC AGC GGC-3′ and 5′-GTG AGC CGG CTG CCC-3′ (Invitrogen) and Taq-DNA polymerase (Promega). dsDNA inserts were then purified, digested with *Bgl*I, purified again, and ligated into a *Sfi*I-digested fUSE5 vector. Select phage clones were analyzed for the presence of insert and confirmed by DNA sequencing.

### Binding assays

Cell phage binding assays were performed as described [27], [28]. A total of 10^6^ cells were incubated in DMEM medium with 10^9^ phage particles of the FLY, FAY or fd-tet phage at room temperature for 2 h. Cell-bound phage was separated from unbound phage by a single centrifugation method named BRASIL (Biopanning and Rapid Analysis of Selective Interactive Ligands) selection [27] and total DNA were extracted with the DNeasy blood and tissue kit as described by the manufacturer (Qiagen). The number of phage particles bound to cells and the quantification of cells DNA were performed by qPhage [28]. For the intermediate filament protein binding assay, recombinant proteins were coated on microtiter (50 µL of 1 mg/mL in PBS) overnight at 4°C. Wells were washed twice with PBS, blocked with PBS containing 3% BSA for 2 h at room temperature, and incubated with 10^9^ phage particles of the individual phage in 50 µL of PBS containing 1.5% BSA. After 3 h at room temperature, wells were washed 10 times with PBS, and phage DNA was extracted with the DNeasy blood and tissue kit as described by the manufacturer (Qiagen) [28].

### Phage *in vivo* homing

All animal experiments were conducted according to protocols approved by the National Institutes of Health Animal Care and Use Committee of the University of Texas MD Anderson Cancer Center (MDACC) and Instituto de Química, Universidade de São Paulo (IQ/USP). Female C57/BL6 mice were obtained from Charles Rivers and housed in the animal facility of the MDACC or bred in house under barrier conditions at the animal care facility of IQ/USP. Mice were anesthetized with 2,2,2-tribromoethanol (Avertin - 0.017 mg/g) and then injected intravenously into the tail vein with 10^10^ FLY, FAY or fd-tet phage particles [19]. Each cohort had three animals and each received a phage clone. After 30 min of phage circulation, mice were perfused through the heart with 10 ml of DMEM. Organs were then weighed and total DNA was extracted with DNeasy blood and tissue kit (Qiagen). The number of bound phage and the DNA content from cells was determined by real-time PCR as described previously [28].

### Fluorescence assays

For immunofluorescence, the cells grown on a 24-well polystyrene plate were washed with PBS two times and when needed were fixed with 2% *p*-formaldehyde in ice-cold PBS for 10 min. The fixed cells were then washed three times with PBS and permeabilized with 0.1% Triton X-100 in PBS for 5 min, washed again with PBS three times, and blocked with PBS with 1% bovine serum albumin for 1 h at room temperature. The cells were incubated with mouse anti-Pan Cytokeratin (1∶1000 dilution) and goat anti-Vimentin (1∶20 dilution) for 1 h at room temperature. After three washes with PBS, the cells were incubated with Alexa-Fluor 488 conjugated to donkey anti-goat IgG or Alexa-Fluor 594-conjugated goat anti-mouse IgG (Invitrogen) at a dilution of 1∶1,000 for 45 min. The cells were washed and counterstained with 1 µM 4,6-diamidino-2-phenylidole (DAPI) before mounting with Vectashield anti-fading medium (Vector Laboratories). The cells were visualized with an Olympus IX-71 inverted fluorescence microscope. Serial images (0.2 µm) were acquired with a Photometrix Cool-SnapHQ charge-coupled device camera driven by DeltaVision software (Applied Precision). Alternatively, serial images (0.2-µm Z-increment) were collected using a 100X objective 1.40 NA using the Cell∧M software (Olympus Europe) in a motorized Olympus IBX81 microscope. All images were obtained using the same acquision parameters (exposure time, minimal and maximal gain). The images were processed by blind deconvolution using Autoquant X 2.1.

## Results

### Bacteriophage as a surrogate of the FLY peptide

**Figure 1:**
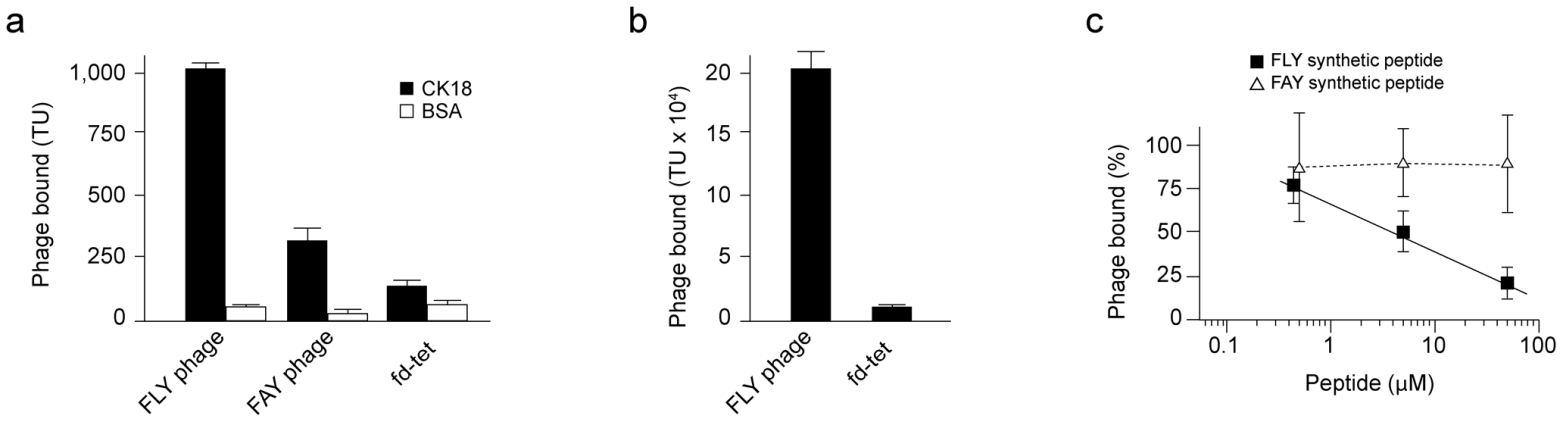
The FLY phage mimics the VTVTNVFLYNRPLN peptide. (a) Binding of FLY phage and fd-tet to immobilized CK18 and to (b) LLC-MK_2_ cells. (c) Binding of FLY phage to CK18 in the presence of increasing concentrations of VTVTNVFLYNRPLN synthetic peptide (FLY, black squares) or the alanine mutagenized version VTVTNVFAYNRPLN synthetic peptide (FAY, open triangles). Results are show as percentage of binding relative to FLY phage in the absence of peptides. Shown are standard error of the mean (SEM) of two biological replicates performed in triplicate.

To generate a filamentous bacteriophage (Fd) displaying the FLY peptide (FLY phage, for short), phage were genetically engineered to express the VTVTNVFLYNRPLN peptide in the outer coat of the virion particle fused to the amino-terminal portion of the pIII minor coat protein. A mutagenized version of the FLY phage was also constructed by substitution of the first leucine residue in the FLY peptide for alanine, previously shown to abolish binding to CK18 [12]. The phage displaying the peptide VTVTNVFAYNRPLN was used as control (FAY phage, for short). To assess whether the FLY phage retains cell and CK18 binding properties, phage binding assays were utilized. Indeed, FLY phage binds to immobilized CK18 (Figure 1a) and to LLC-MK_2_ cells cultured in microtiter wells (Figure 1b). No significant binding of insertless fd-tet or control FAY phage to either CK18 or LLC-MK_2_ cells was observed. To confirm that binding was mediated by the displayed peptide, FLY phage binding to immobilized CK18 was repeated in the presence of increasing concentrations of the cognate synthetic FLY peptide or its mutagenized version (FAY). Only synthetic soluble FLY peptide inhibited phage binding in a dose dependent manner (Figure 1c) and there was no effect of the FAY peptide on FLY phage binding to CK18. In sum, the peptide VTVTNVFLYNRPLN could be successfully displayed on the surface of filamentous phage and the displayed peptide retained CK18 and cell binding properties. Thus, FLY phage can be utilized as a surrogate to study VTVTNVFLYNRPLN peptide interaction with endothelial cells and the vascular bed.

### The FLY peptide binds to endothelial cells in an organ-dependent manner

**Figure 2:**
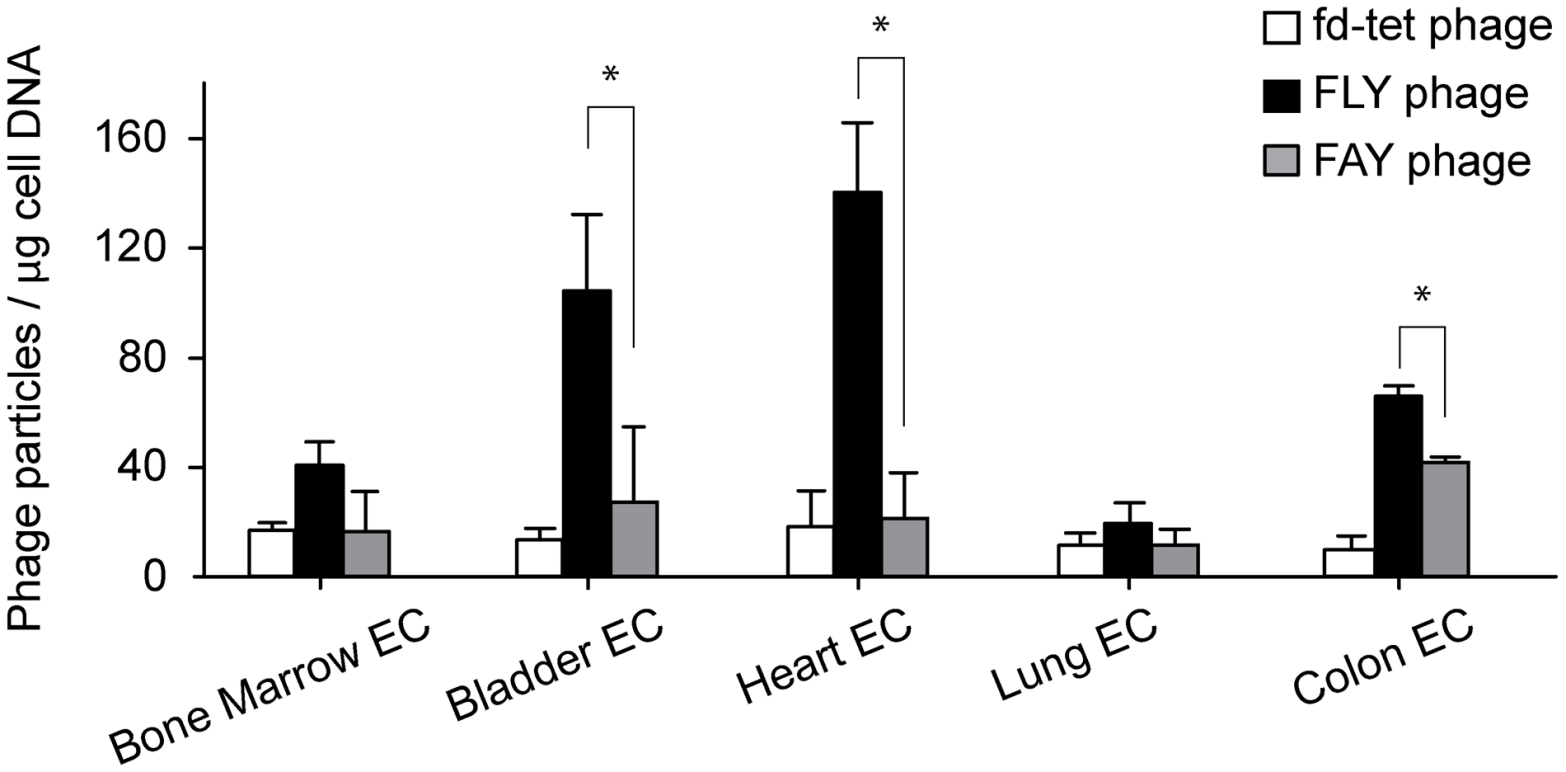
FLY phage binding to organ-derived endothelial cells. Binding of FLY phage to bone marrow, bladder, heart or lung-derived endothelial cells; fd-tet and FAY phage were used as control. Phage binding was normalized to endothelial cell DNA, quantified using ribosomal RNA specific probes. The error bars are standard error of the mean (SEM) of experiments performed in triplicate. Where indicated, * denotes P<0.05.

Having shown that the peptide displayed on the surface of the bacteriophage maintains its biological properties, the FLY phage was then used to evaluate VTVTNVFLYNRPLN role in gp85/trans-sialidase endothelial cell interaction. With that purpose in mind, advantage has been taken of a previously described panel of organ-derived immortalized endothelial cells [21], which have been successfully combined with phage display. A screening on the lung-derived endothelial cell line belonging to this panel led to a peptide that homes the lung vasculature *in vivo* [19]. Here, selection of endothelial cells from this panel was based on tissues to which *T. cruzi* shows selective tropism in humans and animal models: heart, and the digestive and urinary tracts [3], [4], [5], [26]. We thus selected heart, bladder and colon derived endothelial cells. Despite our best efforts, esophagus derived endothelial cells could not be maintained in culture and, therefore, were not used. Lung and bone marrow endothelial cells were included for comparison. The endothelial cells were incubated individually with FLY, FAY of fd-tet phage and cell bound phage separated in a single centrifugation step, as described [27]. The results of two independent experiments showed that FLY phage binds strongly, in dose dependent manner, to endothelial cells derived from two organs in which parasites can be found following infection: heart and bladder [5], [26]. FLY phage also bound, to a lesser extent, to endothelial cells derived from the colon (Figure 2), which is another organ affected by *T. cruzi* infection [4]. In contrast, and as a sign of specificity, FLY phage bound very weakly to bone marrow derived endothelial cells and no significant binding to lung derived cells could be detected. No significant cell phage binding was observed when the cells were incubated with either FAY phage or the fd-tet insertless control phages. These data suggest that the FLY peptide mediates *T. cruzi* interaction with endothelial cells in a tissue and organ selective manner.

### The FLY peptide homes *in vivo*

**Figure 3:**
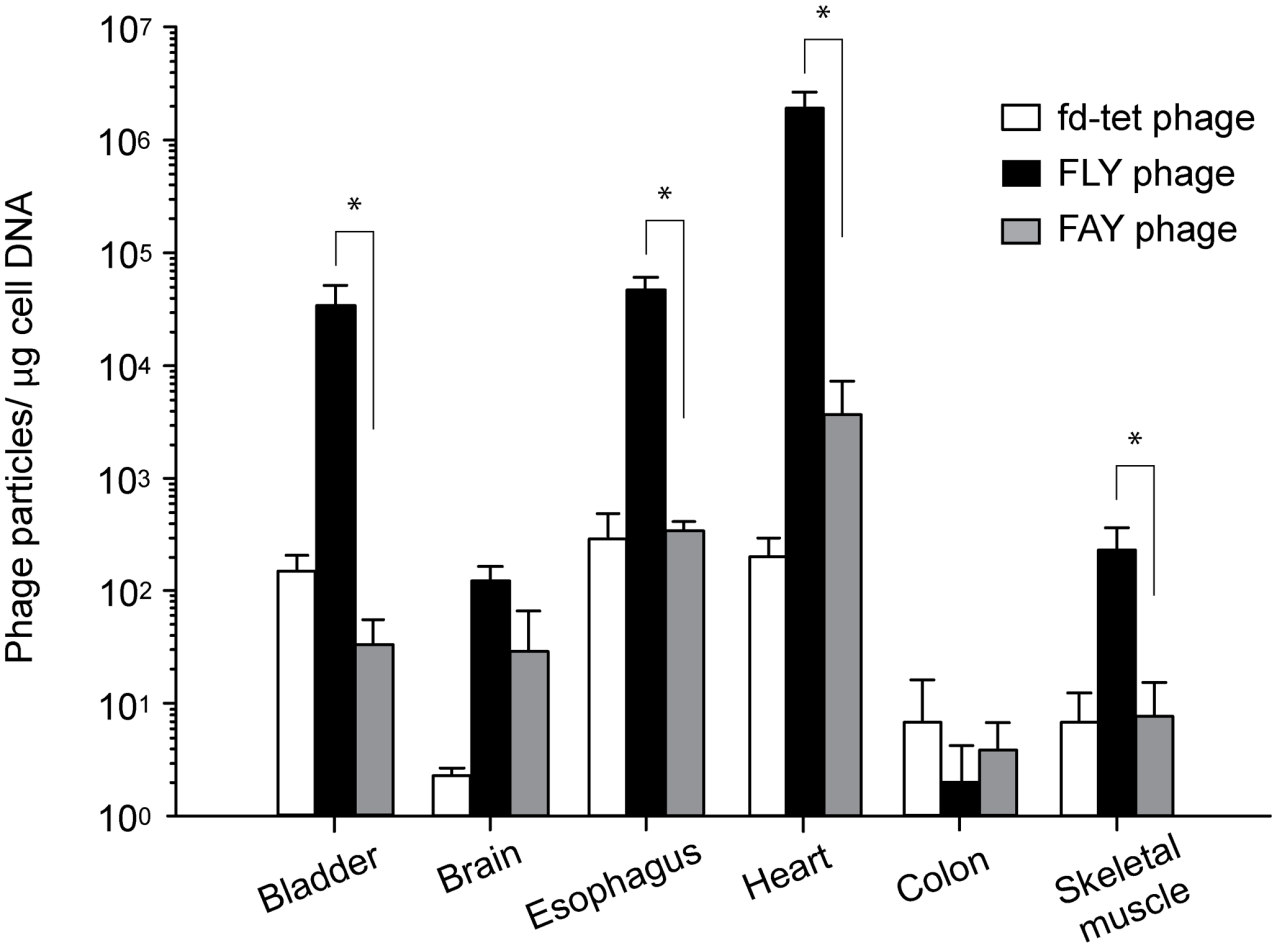
FLY binding to the vasculature of the mouse. FLY and FAY phage homing *in vivo* was evaluated following intravenous phage administration into mice. The number of phage particles that accumulated in the vasculature of select organs was quantified by qPhage. Results are shown relative to the amount of mouse genomic DNA (quantified using ribosomal RNA specific probes) with the SEM of experiments performed in triplicate. Where indicated, * denotes P<0.05.

Phage display *in vivo* is a valuable tool to study vascular ligand-receptor pairs [24]. This approach allows the investigator to probe for receptor expression and accessibility *in vivo* without or with minimal disturbance to the homeostasis of the vascular bed. And because phages are large particles, they remain confined to the circulatory system mimicking the conditions to which *T. cruzi* is exposed while in circulation such as shear-stress forces. To assess whether FLY peptide interaction with the endothelial cells is recapitulated *in vivo*, mice were injected i.v. with FLY-phage and after 30 minutes in circulation the number of phage particles present in different tissues was determined [23], [28]. FLY phage bound to the vasculature of almost all organs analyzed (Figure 3), which again, is in good agreement with the observation that in the early stages of the disease, *T. cruzi* is found in almost every tissue and organ [26], [29]. No significant binding of FAY and fd-tet phages was observed indicating that FLY phage interaction with the vasculature is specific. Noteworthy, however, was the fact that FLY phage bound very strongly to the heart vasculature with an 8,800– fold enrichment when compared to the fd-tet control phage (Figure 3). Significant enrichment of phage particles was also observed with the bladder and esophagus vasculature (213 and 123 times, respectively, relatively to fd-tet), and to a lesser extent, to skeletal muscle endothelium (40 times relative to fd-tet). Megaesophagus is a less common but also important manifestation of Chagas' disease [2], [4]. No significant phage enrichment was detected in the brain, which is in agreement with the lack of disturbances in the high nervous system in patients with Chagas' disease. Interestingly FLY phage did not seem to home *in vivo* to the colon. This is not in agreement with the cell binding data, which showed that FLY phage binds to colon-derived endothelial cells and with the fact that megacolon is the most common manifestation in patients afflicted with Chagas' gastrointestinal disorders. One possible explanation for this conundrum might be related to the number of receptors present in the colon vasculature. The binding of FLY phage to colon endothelial cells was significantly weaker if compared to binding to heart and bladder derived endothelial cells. In sum, phage display *in vivo* data confirmed that FLY phage bind strongly to heart, esophagus and bladder endothelium, which is in good agreement with the results of the cell binding *in vitro* assay and suggest that parasite interaction with the endothelium might be an important contributor to the tropism observed in *T. cruzi* infection.

### The FLY peptide binds to different cytokeratin family members

**Figure 4:**
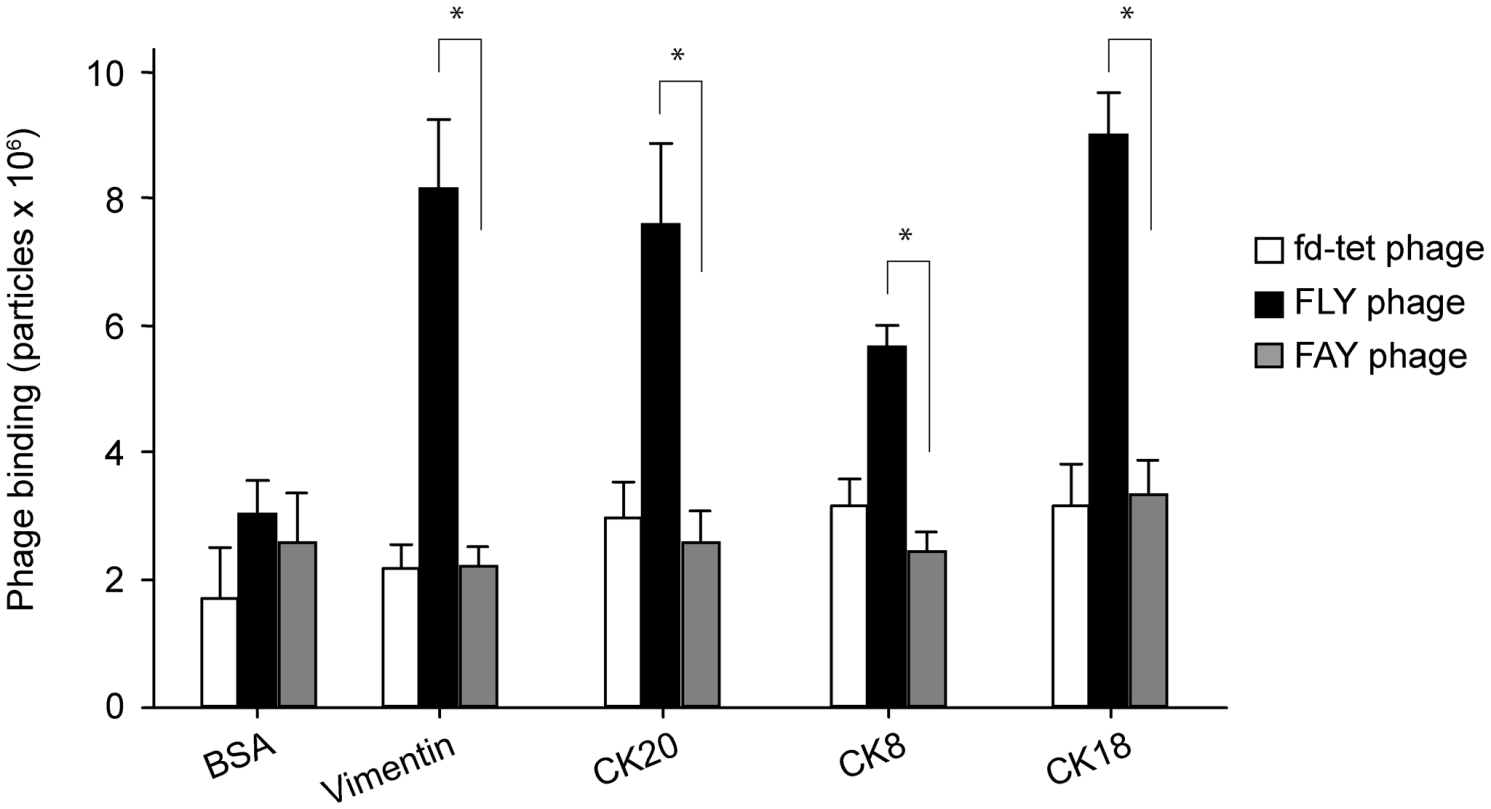
FLY phage interaction with intermediate filament proteins. Phage binding to immobilized cytokeratin-8 (CK8), -18 (CK18) and -20 (CK20), and to vimentin. The FAY and fd-tet phage were used as control. Shown are SEM of experiments performed in triplicate. Where indicated, * denotes P<0.05.

There have been several reports that CK18 is expressed on the surface of cells ([12] and references therein). However, CK18 expression profile is restricted to certain tissues and organs, which is not in good agreement with the almost pan-infective characteristic of *T. cruzi* (the parasite can invade almost any cell line in culture). Because cytokeratins belong to a large multigene family with at least 34 known genes in humans and given the high similarity among the different cytokeratin family members, we asked whether the FLY peptide could interact with distinct cytokeratins. The FLY phage was again used as a surrogate of the peptide and binding to cytokeratin-8 (CK8), expressed by heart muscle cells [30], and CK20, which is abundantly expressed by colon epithelial cells, was performed. Vimentin, another intermediate filament protein expressed by endothelial cells, was also included in the assay. The intermediate filament proteins were immobilized on microtiter plates and incubated with FLY, FAY or fd-tet phage as described in experimental procedures. Interestingly, significant phage binding was observed to all proteins tested (Figure 4). The interaction was specific since it could be blocked by the cognate synthetic FLY peptide but not by its mutagenized soluble version (FAY) (Figure 1c); and no significant binding was observed when FAY or fd-tet control phage were incubated with the immobilized proteins. FLY phage bound more avidly to vimentin and to CK18 or CK20, but it also bound to CK8, although to a lesser extent. No significant binding to BSA was detected. These results suggest that the FLY peptide binds to distinct members of the cytokeratin family and, perhaps, to multiple ligands belonging to the family of intermediate filament proteins.

### Cytokeratins and vimentin are exposed on some endothelial cells

**Figure 5:**
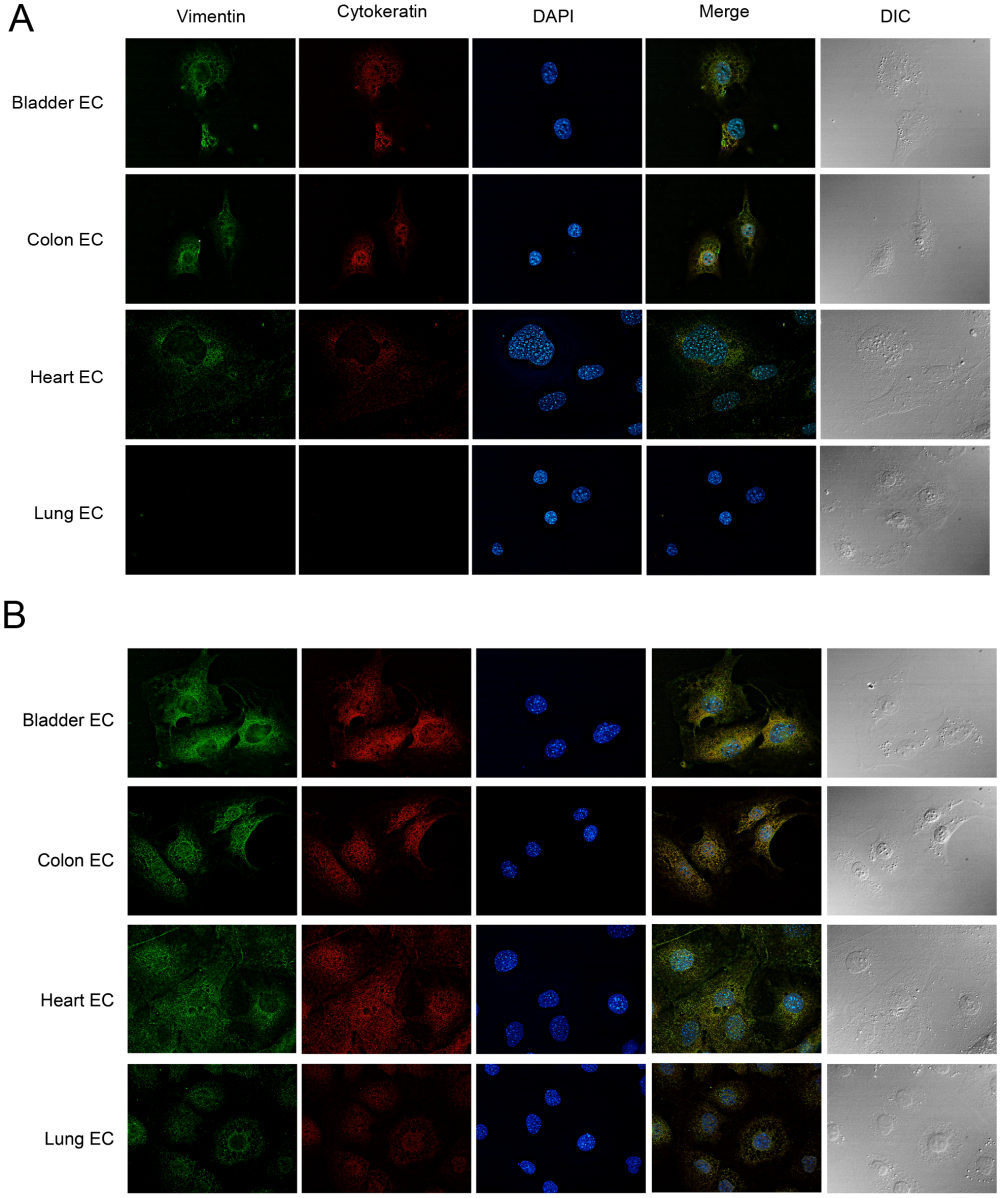
Vimentin and cytokeratin are present on the cell surface. Different organ derived endothelial cells were incubated with anti-pan-CK (red) or anti-vimentin (green) specific antibodies. In (a), antibodies were incubated with live cells and in (b), the antibodies were incubated with p-formaldehyde fixed and detergent permeabilized cells. DAPI staining (blue), merged fluorescence images and the corresponding DIC images are also shown.

Cytokeratins and vimentin are usually recognized as intracellular components of the cytoskeleton, although previous studies have demonstrated the presence of vimentin and cytokeratins on the surface of vascular cells and cardiomyocytes [31]. In order to assess whether these proteins are found on the extracellular milieu, more specifically, on the cell surface of the organ derived endothelial cells used in this study, immunofluorescence experiments were performed. Live cells were incubated with anti-pan cytokeratin or anti-vimentin antibodies. In agreement with the phage binding data, significant labeling with both antibodies were observed for the bladder, heart and the colon endothelial cells but not with the lung derived endothelial cells (Figure 5a). As a further proof of specificity, when cells were previously fixed with *p*-formaldehyde, permeabilized with 0.1% Triton X-100 and then incubated with the antibodies, a strong positive labeling was observed with all cells, including the lung endothelial cells (Figure 5b). These data indicate that bladder, colon and heart derived endothelial cells express CKs and vimentin on the cell surface and further suggest that the preferential binding of FLY-phage to these cells may be due, at least in part, to the presence of extracellular exposed intermediate filament proteins.

## Discussion

The role of the gp85/trans-sialidases in *T. cruzi* infection has been extensively demonstrated by several groups, including ours, although the exact mechanism by which the members of this large gene family mediate parasite cell invasion is still unclear. Different molecules have been identified as putative ligands for gp85/trans-sialidase family members and the pan-specificity toward so many distinct proteins has been explained by the existence of hundreds of gp85/transialidases sharing variable degrees of similarity: different proteins, different ligands. This hypothesis is corroborated by findings, for example, from our group that only a sub-set of acidic gp85/trans-sialidase glycoproteins binds to laminin-1 [8]. However, in contrast with this line of thought, a highly conserved peptide sequence present in all gp85/trans-sialidase family members promotes organ-selective endothelial cell binding, possibly, by interacting with different intermediate filament proteins, in particular, those belonging to the cytokeratin family or to vimentin.

Intermediate filaments are important components of the cytoskeleton, present in nearly every eukaryotic cell [32]. They are comprised of proteins structurally related, including cytokeratins, vimentin and nuclear lamins. The binding of the FLY peptide to different cytokeratins is not unexpected given the high similarity among family members. But the interaction of FLY peptide with vimentin is an interesting finding and suggests that FLY might bind to intermediate filament proteins in general. And since intermediate filament proteins share a common structure and cellular functions [32] but their expression pattern vary, it is possible that *T. cruzi* selected a common binding site shared by different intermediate filament proteins in order to invade a wider variety of cells and tissues. Our data is in agreement with this hypothesis as suggested by the interaction of FLY peptide with CK8 (expressed by muscle cells, epithelial cells), CK20 (epithelial cells) and vimentin (endothelial cells). However, we cannot rule out the participation of other intermediate filament proteins as putative receptors for the parasite because many of these structural proteins have overlapping expression patterns and are often expressed by different cells in multiple tissues. For example, cytokeratin intermediate filaments are heteropolymers formed from equal amounts of type I and type II keratin chains. In fact, the heterodimeric nature of cytokeratin filaments may also explain why parasites still enter cells in which only CK18 expression has been reduced by transient RNA interference [33].

Other studies have already pointed to the presence and importance of cytokeratins in non-epithelial tissues, and more specifically, the heart. Cardiac myocytes express CK8, CK18 and CK19 [30], including the myocardial endothelium compartment [34]; absence of CK19 results in loss of contractile force and myopathy [35]. CK18 and a fragment of CK18 (produced by caspase cleavage) have also been reported as markers for myocardial damage [34], [36], [37]. Interestingly, the caspase cleaved-CK18 fragment seems to accumulate in myocardial lysosomes [34] and because these organelles have a prominent role in parasite cell entering [38], it is tempting to speculate that there might exist a correlation between these two phenomena. In summary, our data point to an important role of intermediate filament proteins in *T. cruzi* cell entering and infection. These proteins, however, are ubiquitously expressed by virtually all eukaryotic cells and new approaches ought to be considered in order to fully appreciate their individual contribution to Chagas' disease.

Finally, using phage as a surrogate and a well established *in vivo* homing assay, we show that the FLY peptide mediates phage distribution to different organs of the mouse with remarkable parallel to the tissue tropism observed in human disease and animal models [3], [4], [5], [39]. Worth mentioning is the strong binding of FLY phage to the heart vasculature, one of the most affected organs in patients with symptoms of chronic Chagas' disease. Almost 70% of the patients show some form of heart dysfunction and die of cardiac problems [3]. These results indicate that the FLY peptide might be an important contributor to tissue tropism, delivering a higher load of parasite to these tissues. It also supports the notion that the vasculature and the endothelial cells are important players in Chagas' disease. Taken together, our data on endothelial cell immortalization and phage display unveiled the important contribution of two large families of proteins, the intermediate filament proteins and the gp85/transialidases, in *T. cruzi* tissue tropism. These data may have important implications in the pathology of Chagas' disease and novel therapeutic approaches for patients afflicted with this disease.
